# Thermal fluid fields reconstruction for nanofluids convection based on physics-informed deep learning

**DOI:** 10.1038/s41598-022-16463-1

**Published:** 2022-07-22

**Authors:** Yunzhu Li, Tianyuan Liu, Yonghui Xie

**Affiliations:** 1 School of Energy and Power Engineering, Xi’an Jiaotong University, Xi’an, 710049 Shaanxi Province People’s Republic of China; 2 Present Address: College of Engineering, Peking University, Beijing, 100089 People’s Republic of China

**Keywords:** Mechanical engineering, Scientific data, Fluid dynamics

## Abstract

Based on physics-informed deep learning method, the deep learning model is proposed for thermal fluid fields reconstruction. This method applied fully-connected layers to establish the mapping function from design variables and space coordinates to physical fields of interest, and then the performance characteristics Nusselt number *Nu* and Fanning friction factor *f* can be calculated from the reconstructed fields. Compared with reconstruction model based on convolutional neural network, the improved model shows no constrains on mesh generation and it improves the physical interpretability by introducing conservation laws in loss functions. To validate this method, the forced convection of the water-Al_2_O_3_ nanofluids is utilized to construct training dataset. As shown in this paper, this deep neural network can reconstruct the physical fields and consequently the performance characteristics accurately. In the comparisons with other classical machine learning methods, our reconstruction model is superior for predicting performance characteristics. In addition to the effect of training size on prediction power, the extrapolation performance (an important but rarely investigated issue) for important design parameters are also explored on unseen testing datasets.

## Introduction

In recent decades, miniaturization technology facilitates the development of higher efficient equipment, such as electronic components, refrigeration, transportation and so on. However, increased performance has created an urgent need for removing the higher heat loads to protect device from high temperature^1^. To resolve this challenge, microchannel heat exchanger (MCHE), known as micro electromechanical systems (MEMS), is widely applied in microscale devices due to its large surface to volume ratios, high convective heat transfer coefficient and smaller size^2^. An obvious fact is that a heat transfer process is determined by the heat transfer device and the utilized working fluid. Thus, the conventional working fluids, such as water and other refrigerant showing scant improvement in thermal properties become one of the limiting factors for higher heat coefficient. The nanofluids combining the base fluid and solid nanoparticles with better thermal conductivity show a promising application in MCHE.

The concept of nanofluids is first proposed by Choi^3^ in 1995, their following works^4,5^ demonstrated that the nanofluids with metallic nanoparticles suspended in conventional heat transfer fluid enhanced heat transfer and reduced pressure drop dramatically. This excellent performance has led to a great upsurge in the study of nanofluids so far. Many state-of-art reviews have been reported on the investigations and applications of nanofluids. Gupta^6^ reviewed the experimental investigations of forced convective heat transfer with different nanofluids. In the review of Ghadimi^7^, the characteristics, numerical model and measurement of thermal conductivity and viscosity were introduced. The thermal and hydraulic performance of nanofluids flowing in mini and micro channels were reported by Sarkar^8^.

As reported above, nanofluids have higher thermal conductivity and corresponding heat transfer than base fluid. However, the heat transfer coefficient shows complicated relationship with not only thermal conductivity but several factors, such as heat capacity, viscosity, flow pattern and so on. Based on numerical and experimental results, many conventional correlations are derived for different operation and nanofluids. In the conventional fitting methods, they suppose that the mapping function from design parameters to target variables satisfies some specific type of function. And then the rest of the fitting work is how to determine the coefficients. This method is simple and convenient, and the precision is acceptable if a proper function is settled. However, the functions between the design variables and objective functions are unknown in most cases, and they are usually selected by experience and numerous attempts. The selection process of functions becomes laborious if massive design parameters are considered or the relationship shows strong nonlinear.

Recently, more and more researchers adopt machine learning methods, such as adaptive neuro-fuzzy inference system^9^, least squares support vector machine^10^, Gaussian process regression^11^, artificial neural network (ANN)^12–15^ and so on, to predict thermophysical properties and thermodynamic performance for nanofluids. The advantages of those machine learning methods include: no specific type of function should be supposed in advance; more design parameters can be included; higher calculation efficiency for prediction; and more importantly, the fitting ability is stronger for nonlinear functions. However, these approaches of conventional fitting methods or machine methods overlooked a fact that most target variables, such as the thermodynamic performance, can be regarded as a kind of refinement from physical fields. Those methods only focus on the mapping functions between design variables and some target variables, which cause the lack of physics interpretability and limit their scope of application. Different from these traditional machine learning methods, we propose a reconstruction framework utilizing deep neural network (DNN) to reconstruct physical fields instead of thermodynamic performance in this study. Once the physical fields are reconstructed, any interested thermodynamic performance can be extracted from fields directly.

As the most well-known methods in supervised learning, the neural network was demonstrated to approximate any function with sufficiently large and deep network by the universal approximation theorem in 1989^16^. Recently, according to the widespread growth of data and the rapid advances of supercomputer, the power and flexibility of DNNs have led to a series of breakthroughs for computer vision, natural language processing and many other directions of artificial intelligence. In thermal and fluid mechanics, many complex tasks, such as super-resolution reconstruction^17–19^, turbulence model improvement^20–22^, field reconstruction^23–27^, flow control^28^, design and optimization^29,30^ and so on, can be accomplished impressively with deep learning architectures.

Traditionally, the physical fields are obtained by means of experiments measuring physical variables in limited space or numerical simulations solving conservation equations. Despite significant advances in experiments and numerical simulations in recent decades, the progress of experiments and simulations is still time-consuming, laborious and need prior experience. The advantages of DNN enables physical field prediction quickly without manual intervention. Mostly, the physical fields are treated as images (spatial data) or videos (spatial–temporal data), and then many prediction or reconstruction models inspired by computational vision^31–33^ are reported. Guo^23^ proposed an approximation model with encoder and decoder to predict flow field from geometry and boundary representations based on DNN. Hennigh^24^ proposed Lat-Net to compress numerical simulations obtained by Lattice Boltzmann Method using convolutional neural network (CNN). Lat-Net is composed of three parts, an encoder to compress the state of simulations, a compression mapping to learn the dynamics on compressed state and a decoder realizing the decompress process. Lee^34^ utilized a multi-scaled generative adversarial network (GAN) to predict time series of laminar vortex shedding over a cylinder based on previous fields. Kim^35^ synthesized discrete velocity fields in space and time from a set of reduced parameters. In our previous study^36,37^, a reconstruction model with GAN and fields gradient loss is firstly proposed to predict the physical fields of nanofluids microchannel based on design variables, limited measurement and the effect of training size, measuring uncertainty and measuring layouts are discussed in detail. The image-inspired reconstruction models can obtain the overall physical fields in one prediction at the millisecond level and capture the spatial correlations among grid points efficiently. Despite great potential, practical implementation applying CNN models to computational or experimental fluid dynamics remains limited. Firstly, the field reconstruction task realized by CNN models is still a black box without physics interpretability, and the successes of CNN models mainly rely on the powerful feature extraction and the ability to resolve nonlinear problems. In essence, the thermal and fluids examples are driven by prior knowledge of conservation laws. Secondly, due to the special convolution operations on feature, the input or output fed to CNN model should be preprocessed to structure grid data. In addition to some examples with simple geometry or structure whose grids can be easily transformed to required structured data^38^, the physical fields of most thermal and fluids examples with complicated geometry should be interpolated to designed structured grid points, which may cause the lack of information around large gradience. Besides, the definition of default values in areas without fields information is a pending problem.

For thermal and fluid mechanics, there exists an obvious prior physical knowledge, which is conservation principles, and all available data respect the physical laws given by conservation principles. Many trials have been made to incorporate and enforce known flow physics in applications of DNN. Zhao^39^ combined domain knowledge and ANN to predict the critical heat flux. In the multi-scaled GAN constructed by Lee^34^, the conservation principles are formulated using the triangle inequality to approximate to the original forms and the loss function of conservation principles is minimized to compare the difference between predicted fields and ground truth. However, the introduction of physical informed loss function requires the pre-designed nontrainable convolutional filters^38^ or loss equations around the whole fields^34,35^, and it heavily depends on the mesh information. Recently, physics-informed neural networks (PINNs) introduced by Karniadakis and Raissi^40,41^ that are trained to solve supervised tasks respecting physical laws are introduced. According to their explorations of physics informed neural network on surface breaking crack identification^42^, biological reactions^43^, compartmental disease transmission models^44^ vortex-induced vibrations^45^, heat transfer problems^46^ and other flow mechanism^47^, the physics conservation principles can be utilized to be loss function and regularization term with the automatic differentiation and the back-propagation mechanism, which can be essentially regarded as prior knowledge to constrain the space of admissible solutions. Another advantage is physics-informed neural networks can provide a mesh-free solver as the discrete interpolators in both space and time over the computational domain, which can efficiently handle the unstructured mesh of any numerical methods. Thus, the possibility of using PINNs to approximate flow in idealized stenosis^48^, arterial blood pressure^49^ and high-speed flow^50^ are investigated. Moreover, Karniadakis^51^ extended PINNs (XPINNs) to space–time domain decomposition for nonlinear partial differential equations in arbitrary complex-geometry domains. Based on XPINNs and another extension (namely the conservative PINNs^52^), a distributed framework for PINNs^53^ is proposed with several advantages, such as parallelization capacity, large representation ability, efficient hyperparameter tuning and is particularly effective for multi-scale and multi-physics problems.

To the best of authors’ knowledge, this is the first attempt of applying PINNs on the field reconstruction for nanofluids convection problem. In this study, we applied PINNs to reconstruct physical fields (pressure, temperature and velocity) of nanofluids convection from varying design variables, including Reynold number, nanofluids properties, geometric parameters and boundary conditions. The primary contributions of this work are listed as followed:

1. The physics-informed and mesh-free prediction model for nanofluids convection in microchannels is proposed to reconstruct all interested physical fields and then extract the heat transfer characteristics (*Nu* and *f* for instance) directly.
2. This method enforces the conservation laws by introducing mass, momentum and energy continuity equations to guide the training of deep learning neural network.
3. To evaluate the accuracy of our model in the sense of theory and engineering, the performance of reconstructed fields, conservational residual and the heat transfer and flow characteristics interested in engineering are discussed in detail.
4. Except for the effect of training size, we also focus on an important but lack of attention issue, that is the extrapolation ability of the neural network on the unknown domains.

The main context of this paper is organized as followed: In section B, the overall architecture of applied physics informed neural network is presented and the data set of nanofluids convection applied to train and test reconstruction network is described. Next, the prediction performance is analyzed for physical field visualizations. Detailed physical fields distributions, evaluation criteria and performance are conducted in section C. And then the comparisons of our method and other surrogate models on the prediction performance of performance characteristics are presented. Moreover, the effect of training size and the extrapolation performance are investigated in the latter part of section C. Finally, the conclusions are summarized in section D.

## Methods

### Overall architecture

In physics-informed neural network, the mathematical physics is enforced as partial differential equations (PDE) or ordinary differential equations. The steady governing equations for heat transfer and flow of nanofluids can be expressed as Eq. (1), including the mass, momentum and energy conservation principles.

1 $${\mathbf{\mathcal{N}}}({\mathbf{x}},{{\varvec{\uptheta}}}) = {\mathbf{0}}: = \left\{ {\begin{array}{*{20}c} {\nabla \rho {\mathbf{u}} = 0} \\ {({\mathbf{u}} \cdot \nabla )\rho {\mathbf{u}} - \nabla \cdot (\mu \nabla {\mathbf{u}}) + \nabla p + b_{f} = {\mathbf{0}}} \\ {({\mathbf{u}} \cdot \nabla )c_{p} \rho t - \nabla \cdot (\lambda \nabla t) - s_{t} = 0} \\ \end{array} } \right. \, {\mathbf{x}} \in {{\varvec{\Omega}}} \subset {\mathbb{R}}^{{D_{{\mathbf{x}}} }} ,{{\varvec{\uptheta}}} \in {\mathbb{R}}^{{D_{{{\varvec{\uptheta}}}} }}$$

where ${{\mathcal{N}}}(\bullet)$ are the nonlinear PDE operators representing the conservation principles, **x** is the space coordinate, **θ** is a state parameters set to describe the physical system such as fluid properties, boundary conditions, and geometry of the domain, which can be expressed as a *D*_**θ**_-dimensional vector; *ρ* and *ν* represent density and viscosity of the fluid, respectively; *b*_*f*_ is the body force; and **Ω** denotes the fluid computational domain. The velocities **u**, pressure *p* and temperature *t* can be regarded as functions of the space coordinate **x**, and variable parameters **θ**. Specifically, the physical fields can be uniquely determined when suitable boundary conditions are prescribed,

2 $${\mathcal{B}}{(}{\mathbf{x}},{{\varvec{\uptheta}}}{)} = {\mathbf{0}} \quad \, {\mathbf{x}} \in \partial \Omega ,{{\varvec{\uptheta}}} \in {\mathbb{R}}^{{D_{{{\varvec{\uptheta}}}} }}$$

where ${\mathcal{B}}$ is the general differential operators that define the boundary conditions and ∂Ω denotes the boundary regions. Given a set of specific parameters **θ**, the mapping functions of physical fields, i.e. **u**(**x**), *p*(**x**) and *t*(**x**), can be obtained by discretizing corresponding governing equations in the Eq. (1) using numerical methods, such as Finite Difference /Finite Volume /Finite Element methods. However, the numerical process requires time-consuming mesh generation and iteratively solving large linear/nonlinear systems. Due to the tedious regeneration of computational mesh, the traditional numerical methods become more challenging with diverse geometries involved.

Different from the traditional numerical methods, the PINNs simply focuses on the reconstruction of physical scalars for single temporal and spatial point rather than the whole physical fields in one prediction. This strategy enables the deep neural network solving the conservation principle quickly without the trouble of mesh generation. To describe the process of numerical method and reconstruction model, some mathematic expressions are listed as follows.

3 $${\hat{{\varvec{\uppsi}}}} = \{ \hat{p}{,}{\hat{\mathbf{u}}}^{T} {,}\hat{t}\}^{T} = {\tilde{\mathbb{F}}}{(}{\mathbf{x}}{,}{{\varvec{\uptheta}}}{;}\Theta {)} \approx {\mathbb{F}}{(}{\mathbf{x}}{,}{{\varvec{\uptheta}}}{) = }\{ p{,}{\mathbf{u}}^{T} {,}t\}^{T} { = }{{\varvec{\uppsi}}}$$

As shown in Eq. (3), the training data is obtained by the traditional numerical or experimental methods based on design parameters **θ** and coordinates **x**, and the acquisition method of raw physical fields ${{\varvec{\uppsi}}} = \{ p{,}{\mathbf{u}}^{T} {,}t\}^{T}$ is considered as a mapping ${\mathbb{F}}{(}{\mathbf{x}}{,}{{\varvec{\uptheta}}}{)}$. Correspondingly, the well-trained deep neural network model constructs an approximate mapping $\widetilde{\mathbb{F}}({\varvec{x}},{\varvec{\theta}};\Theta )$ with learnable parameters $\Theta$ substituting for the traditional method ${\mathbb{F}}{(}{\mathbf{x}}{,}{{\varvec{\uptheta}}}{)}$ to reconstruct physical fields ${\hat{{\varvec{\uppsi}}}} = \{ \hat{p}{,}{\hat{\mathbf{u}}}^{T} {,}\hat{t}\}^{T}$. According to the description of the heat transfer and flow problem in Eq. (1–2), the key point of reconstructing physical fields is trying to conform with the nonlinear PDEs ${{\mathcal{N}}}({\mathbf{x}},{{\varvec{\uptheta}}}) = {\mathbf{0}}$ and differential operator ${\mathcal{B}}{(}{\mathbf{x}},{{\varvec{\uptheta}}}{)} = {\mathbf{0}}$. Thus, the cost function of the model is considered as the combination shown in Eq. (4). Remarkably, as described in Eq. (5), the training process of the model can be regarded as the optimization process of the proper learning parameters $\Theta^{*}$ with the cost function ${\mathcal{L}}$.

4 $${\mathcal{L}}{(}{\tilde{\mathbb{F}}}{;}\Theta {)} = \left\| {{{\mathcal{N}}}({\mathbf{x}},{{\varvec{\uptheta}}})} \right\|_{\Omega } + \left\| {{\mathcal{B}}({\mathbf{x}},{{\varvec{\uptheta}}})} \right\|_{\partial \Omega }$$

5 $$\Theta^{*} = \mathop {\arg \min }\limits_{\Theta } {\mathcal{L}}{(}{\tilde{\mathbb{F}}}{;}\Theta {)}$$

### Nanofluids heat convection problem

**Figure 1:**
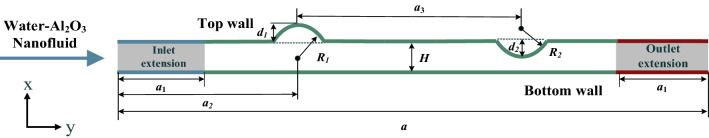
Diagram of the physical configuration.

In this study, a two-dimensional convection problem for the water-Al_2_O_3_ nanofluids in microchannels are validated regarding to this physics informed neural network model. Though the application of nanofluids become engineering gradually and the application scenario becoming more and more complex, the fundamental researches on the heat and flow behavior in simplified microchannel is still important and necessary. Thus, a simple microchannel model as depicted in Fig. 1 is studied. The microchannel is composed by top wall and bottom wall, where the bottom wall is a smooth plate with infinite length while the top wall is a roughed infinite plated with two grooves/protrusions. The total length of the microchannel is *a* and the height is *H*. The inlet and outlet are extended to ensure fully developed and prevent the nanofluids from flowing backward. The lengths of extensions are both *a*_1_. The locations of two grooves/protrusions are determined by the interval of *a*_3_ and the length between the inlet and first groove/protrusion *a*_2_. And the geometries of two grooves/protrusions are defined by two parameters: radius *R*_1_ and *R*_2_, and relative depth *δ*_1_ = *d*_1_/*R*_2_ and *δ*_2_ = *d*_2_/*R*_2_. It should be noticed that the depth of the groove is positive *d* > 0 while the depth of protrusion is negative *d* < 0. Five geometric parameters related to the location and shapes of grooves/protrusions, including interval of *a*_3_, radius *R*_1_ and *R*_2_, and relative depth *δ*_1_ and *δ*_2_, are set as design variables and the other geometric constants are listed in Table 1.

**Table 1:** The list of geometric constants.

Geometric constants	*H*/μm	*a*/μm	*a*_1_/μm	*a*_2_/μm
Value	200	5000	750	1750

The water-Al_2_O_3_ nanofluids is composed of base fluid water and nanoparticles Al_2_O_3_ with a diameter of 30 nm at a certain proportion. In practice, the heat transfer and flow problem for nanofluids is a kind of multiphase flow. To simplify the simulation, the following assumptions are made: the nanoparticles are all spheres with a diameter of 30 nm and distributed uniformly in the base fluid, so the two-phase nanofluids can be equivalent to a single-phase fluid. The thermo-physical properties of base-fluid water and nanoparticle Al_2_O_3_ are shown in Table 2, and thermo-physical properties nanofluids are calculated as followed.

**Table 2:** Physical properties of materials at 293 K and atmosphere.

Material	Density/kg∙m^−3^	Specific heat/J∙kg^−1^∙K^−1^	Thermal conductivity/W∙m^−1^∙K^−1^	Dynamic viscosity/Pa∙s
Water	998.2	4182.0	0.597	9.93 × 10^–4^
Al_2_O_3_	3880.0	773.0	36.0	\

The density of applied nanofluids *ρ*_n_ is calculated as:

6 $$\rho_{{\text{n}}} = (1 - \varphi /100)\rho_{{\text{b}}} + \varphi \cdot \rho_{{\text{p}}} /100$$

where *φ* denotes the volume fraction of nanofluids, *ρ*_b_ and *ρ*_p_ represent the density of base fluid water and nanoparticles Al_2_O_3_, respectively. The specific heat capacity^54^ of applied nanofluids *Cp*_n_ is calculated as:

7 $$Cp_{{\text{n}}} = [(1 - \varphi /100)Cp_{{\text{b}}} \rho_{{\text{b}}} + \varphi Cp_{{\text{p}}} \rho_{{\text{p}}} /100]/\rho_{{\text{n}}}$$

where $Cp_{{\text{b}}}$ and $Cp_{{\text{p}}}$ represent the specific heat capacity of base fluid water and nanoparticles Al_2_O_3_, respectively. The thermal conductivity^55^ of nanofluids *λ*_n_ is defined as:

8 $$\lambda_{{\text{n}}} = 0.25[(3\varphi /100 - 1)\lambda_{{\text{p}}} + (2 - 3\varphi /100)\lambda_{{\text{b}}} + \sqrt \Delta$$

where *λ*_b_ and *λ*_p_ represent the specific heat capacity of base fluid water and nanoparticles Al_2_O_3_, and $\Delta$ is shown as below:

9 $$\Delta = [(3\varphi /100 - 1)\lambda_{{\text{p}}} + (2 - 3\varphi /100)\lambda_{{\text{b}}} ]^{2} + 8\lambda_{{\text{p}}} \lambda_{{\text{b}}}$$

The dynamic viscosity coefficient^1^ of nanofluids $\mu_{{\text{n}}}$ is formulated as

10 $$\mu_{{\text{n}}} = \mu_{{\text{b}}} [123(\varphi /100)^{2} + 7.3\varphi /100 + 1]$$

where $\mu_{{\text{b}}}$ dynamic viscosity coefficient of water.

Considering the infinite microchannel studied in this work, the calculation models are simplified to two-dimensional models. Thus, mathematic expression of conservation equations for incompressible nanofluids in Eq. (1) can be written as:

11 $${{\mathcal{N}}}({\mathbf{x}},{{\varvec{\uptheta}}}) = {\mathbf{0}}: = \left\{ {\begin{array}{*{20}c} {\frac{\partial u}{{\partial x}} + \frac{\partial v}{{\partial y}} = 0} \\ {u\frac{\partial u}{{\partial x}} + v\frac{\partial u}{{\partial y}} + \frac{1}{{\rho_{{\text{n}}} }}\frac{\partial p}{{\partial x}} - \frac{{\mu_{{\text{n}}} }}{{\rho_{{\text{n}}} }}\left( {\frac{{\partial^{2} u}}{{\partial x^{2} }} + \frac{{\partial^{2} u}}{{\partial y^{2} }}} \right) = 0} \\ {u\frac{\partial u}{{\partial x}} + v\frac{\partial u}{{\partial y}} + \frac{1}{{\rho_{{\text{n}}} }}\frac{\partial p}{{\partial x}} - \frac{{\mu_{{\text{n}}} }}{{\rho_{{\text{n}}} }}\left( {\frac{{\partial^{2} u}}{{\partial x^{2} }} + \frac{{\partial^{2} u}}{{\partial y^{2} }}} \right) = 0} \\ {u\frac{\partial t}{{\partial x}} + v\frac{\partial t}{{\partial y}} - \alpha_{{\text{n}}} \left( {\frac{{\partial^{2} t}}{{\partial x^{2} }} + \frac{{\partial^{2} t}}{{\partial y^{2} }}} \right) = 0} \\ \end{array} } \right. \,$$

where *u* and *v* are the velocity along *x* and *y* coordinate; *p* and *t* represent temperature and pressure quantities; $\alpha_{{\text{n}}} { = }\lambda_{{\text{n}}} /\rho_{{\text{n}}} Cp_{{\text{n}}}$ is defined as the thermal diffusion coefficient.

The numerical simulations are all obtained by solving conservation equations with the commercial numerical software FLUENT. To confirm the simulation accuracy, the SIMPLE algorithm is adopted to couple pressure and velocity item and the calculation domains are discretized by the second-order upwind scheme. As listed in Eq. (14–15), the inlet condition is set as velocity input and the inlet temperature of nanofluids is defined at 293 K; the non-slip wall condition is imposed on both up and down walls and the same constant heat flux is set for top and bottom walls.; the outlet pressure of atmosphere is utilized.

12 $${\mathcal{B}}({\mathbf{x}},{{\varvec{\uptheta}}}) = {\mathbf{0}}: = \left\{ {\begin{array}{*{20}c} {p|_{x = L} = 0} \\ {u|_{x = 0} - u_{\infty } = 0;v|_{x = 0} = 0} \\ {u|_{{\text{top;bottom}}} = 0;v|_{{\text{top;bottom}}} = 0} \\ {t|_{x = 0} - 293 = 0; = \frac{\partial t}{{\partial {\mathbf{n}}}}|_{{\text{top;bottom}}} - q = 0} \\ \end{array} } \right.$$

**Figure 2:**
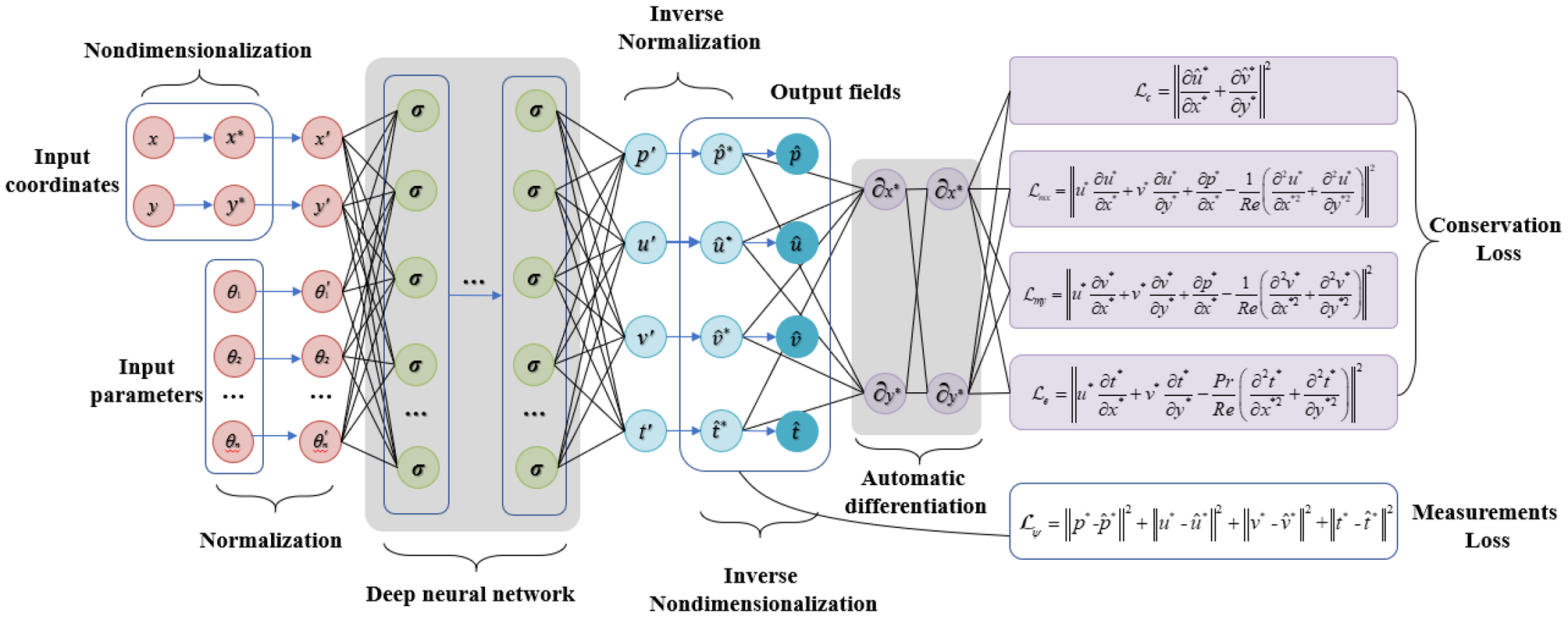
The overall architecture.

Reynold number *Re* is defined as:

13 $$Re = \frac{{\rho_{{\text{n}}} u_{\infty } D_{{\text{h}}} }}{{\mu_{{\text{n}}} }} = \frac{{2\rho_{{\text{n}}} u|_{x = 0} H}}{{\mu_{{\text{n}}} }}$$

where the hydraulic diameter *D*_h_ is 2*H*, and $u_{\infty }$ is the inlet velocity. The mesh generations for all cases are performed by software ICEMCFD. As shown in Fig. 2, the structured grids are divided into the whole computational domain and the grids around grooves/protrusions are refined. According to the physical geometry, the mesh model is composed of three parts: inlet extension, outlet extension and reconstruction region which is utilized as training and testing datasets.

### Dataset description

To embrace the physics information as much as possible, about 6000 cases with eight design variables for the water-Al_2_O_3_ nanofluids in the microchannel including properties of nanofluids, geometric parameters and boundary conditions are sampled by Latin Hypercubic Sampling method. The raw dataset is divided into two parts: the training dataset (50%, 3000 cases) and the testing dataset (50%, 3000 cases).

The configurations and layouts of two protrusions and grooves are important for heat transfer and flow behavior. Thus, the geometric parameters including radium *R*_1_/*R*_2_, relative depth *δ*_1_/*δ*_2_ of grooves/protrusions and the interval length *a*_*3*_ between two grooves/protrusions are taken as design variables. Besides, the volume fraction *φ* of nanofluids is set as one of the design variables because it becomes a determining factor for the thermo-physical properties once the nanoparticle is determined. Finally, the boundary conditions including Reynold number *Re* indicating the inlet velocity and the heat flux *q* imposed on top and bottom walls are also taken into consideration. All the design variables and their ranges are listed in Table 3.

**Table 3:** Varying scope for design parameters.

Design variables	*Re*	*φ*/%	*a*_3_/μm	*R*_1_/μm	*R*_2_/μm	*δ*_1_	*δ*_2_	*q*/W∙m^−2^
Lower limit	10	0.1	30	5	5	− 0.8	− 0.8	100,00
Upper limit	1000	10	150	35	35	0.8	0.8	1,000,00

Apparently, each case for microchannel with nanofluids is determined by design variables ${\varvec{\theta}}=\{{\theta }_{1},{\theta }_{2},\cdots ,{\theta }_{{D}_{{\varvec{\theta}}}}{\}}^{T}$, which is ${\varvec{\theta}}=\{Re, \varphi , {a}_{3},{R}_{1},{R}_{2},{\delta }_{1},{\delta }_{2},q{\}}^{T}$ in this study. For each case, the computational domain is divided by 800 × 40 grid points and only 550 × 40 grid points containing main heat transfer and flow characteristics in the reconstruction region are used. Every grid point is considered as a sample point located by corresponding coordinates ${\varvec{x}}=\{x, y{\}}^{T}$ for training and testing dataset. Therefore, the input for a sample point is the combination of design variables and coordinates ${\varvec{\xi}}=\{{{\varvec{x}}}^{T}, {{\varvec{\theta}}}^{T}{\}}^{T}$ and the output is the interested physical quantities ${\varvec{\psi}}=\{p,u,v,t{\}}^{T}$, that is pressure p, temperature t and velocity attributes along two coordinates *u* and *v*.

With 550 × 40 sample points in each case, there are 132,000,000 sample points can be collected in all 6000 cases. The input of the training dataset is represented as ${\mathcal{D}}_{{\varvec{\xi}}}^{N}=\{{{\varvec{\xi}}}_{1},{{\varvec{\xi}}}_{2},\dots ,{{\varvec{\xi}}}_{N}\}$, while the output physical fields can be defined as ${\mathcal{D}}_{{\varvec{\psi}}}^{N}=\{{{\varvec{\psi}}}_{1},{{\varvec{\psi}}}_{2},\dots ,{{\varvec{\psi}}}_{N}\}$. The number of sample points in training dataset is ${N}_{s}=\mathrm{66,000,000}$ for 3000 cases. It should be emphasized that 3000 cases for training are selected randomly and then all the sample points are disrupted together. In the testing process, all the sample points in one specific case with same design variables should be taken as input to predict the whole physical fields.

### Implementation of deep learning model

Focusing on the nanofluids convection, we establish the deep learning model as shown in Fig. 2 by deep neural network. To ensure the network convergence, the nondimensionalization and normalization methods are involved to scale the input and output tensor. Thus, the input coordinates should be nondimensionalized and normalized while the input parameters of governing equations just need to be normalized. After subsequent operations of deep neural network, interested physical quantities experienced nondimensionalization and normalization operations are predicted. Generally, the measurement loss of the difference between true and predicted normalized physical scalars (the output of DNN after inverse normalization) along with proper optimization algorithm can be utilized to drive the training process of DNN. However, for incorporating the first principle, the conservation loss constructed based on normalized governing equations are required by leveraging automatic difference mechanism. If the output fields are need, then an inverse nondimensionalization should be conducted after the inverse normalization. In the following, we introduce the nondimensionalization and normalization methods applied, the detailed network structure and loss function, and the learning strategy employed in this deep learning framework.

#### Nondimensionalization and normalization

The order of magnitude of the different physical quantities, pressure, velocities, temperature have a significant relative difference, e.g., *p* ∼ 10^4^ Pa, *u* ∼ 1 m/s, *v* ∼ 10^–1^ m/s and *t* ∼ 10^2^ K, which casts great difficulty on the training of the neural network. The significant difference in magnitude of the parameters creates a systematic problem for the training of the physics-informed neural network, as this difference in scales will have a severe impact on the magnitude of the back-propagated gradients that adjust the neural network parameters during training. To overcome this problem, we employ a nondimensionalization and normalization technique with the purpose of scaling the input and the output of the neural networks in a proper scale (e.g., (p^*^, t^*^, u^*^, v^*^) ∼ *O*(1)) and normalizing the spatial and temporal coordinates to have zero mean and unit variance for training the neural networks more efficiently. Although there could be a way to weight the components of the loss function to mitigate the bias casted into the loss function due to this discrepancy across scales, this process would require a lot of guess-work and tuning. On the other hand, the proposed nondimensionalization strategy achieves the goal of normalizing the variables in a physically justified and intuitive manner that adheres to the requirements of standard neural net initialization strategies (e.g., Xavier initialization) and yields a robust workflow that is free from ad-hoc hacks and guesswork. For the purpose of nondimensionalization we introduce some characteristic variables, which are commonly used in multi-scale physics modeling^50^ in order to simplify the equations. For this problem the characteristic length *H* and the characteristic velocity *U*_*∞*_. are applied. Thus, u^*^ and v^*^ are the non-dimensional velocity along *x* and *y* coordinate; p^*^ and t^*^ represent non-dimensional pressure and temperature quantities. The non-dimensional quantities are defined as following:

14 $$x^{*} { = }\frac{x}{H},y^{*} { = }\frac{y}{H},p^{*} = \frac{p}{{\rho_{n} u_{\infty }^{2} }},u^{*} { = }\frac{u}{{u_{\infty } }},v^{*} { = }\frac{v}{{u_{\infty } }},t^{*} = \frac{{t_{w} - t}}{\Delta t}{ = }\frac{{qH/\lambda_{n} + t_{f} - t}}{{qH/\lambda_{n} }}$$

Substituting the Eqs. (14) into the Eq. (11), then the conservation equations can be simplified as Eq. (15).

15 $$\left\{ {\begin{array}{*{20}c} {\frac{{\partial u^{*} }}{{\partial x^{*} }} + \frac{{\partial v^{*} }}{{\partial y^{*} }} = 0} \\ {u^{*} \frac{{\partial u^{*} }}{{\partial x^{*} }} + v^{*} \frac{{\partial u^{*} }}{{\partial y^{*} }} + \frac{{\partial p^{*} }}{{\partial x^{*} }} - \frac{1}{Re}\left( {\frac{{\partial^{2} u^{*} }}{{\partial x^{*2} }} + \frac{{\partial^{2} u^{*} }}{{\partial y^{*2} }}} \right) = 0} \\ {u^{*} \frac{{\partial v^{*} }}{{\partial x^{*} }} + v^{*} \frac{{\partial v^{*} }}{{\partial y^{*} }} + \frac{{\partial p^{*} }}{{\partial x^{*} }} - \frac{1}{Re}\left( {\frac{{\partial^{2} v^{*} }}{{\partial x^{*2} }} + \frac{{\partial^{2} v^{*} }}{{\partial y^{*2} }}} \right) = 0} \\ {u^{*} \frac{{\partial t^{*} }}{{\partial x^{*} }} + v^{*} \frac{{\partial t^{*} }}{{\partial y^{*} }} - \frac{Pr}{{Re}}\left( {\frac{{\partial^{2} t^{*} }}{{\partial x^{*2} }} + \frac{{\partial^{2} t^{*} }}{{\partial y^{*2} }}} \right) = 0} \\ \end{array} } \right. \,$$

The Prandtl Number *Pr* applied in governing equations are shown as below:

16 $$Pr = \frac{{\mu_{{\text{n}}} Cp_{{\text{n}}} }}{{\lambda_{{\text{n}}} }}$$

After the dimension, eight design variables and two coordinates are considered as input. However, the distributions for different variables show a large deviation. Thus, the Z-score normalization method^56^ is utilized to scale input. The formulation of the normalization method for each sample point is:

17 $${{ \{ }}{\mathbf{x^{\prime}}}^{T} {,}{\mathbf{\theta^{\prime}}}^{T} {{\} }}^{T} { = }{{\varvec{\upxi}}}^{\prime}{ = (}{{\varvec{\upxi}}} - {\mathbb{E}}_{{{{\varvec{\upxi}}} \sim \user2{\mathcal{D}}_{{{\varvec{\upxi}}}}^{N} }} [{{\varvec{\upxi}}}])/\sqrt {{\mathbb{V}}_{{{{\varvec{\upxi}}} \sim \user2{\mathcal{D}}_{{{\varvec{\upxi}}}}^{N} }} [{{\varvec{\upxi}}}]}$$

where ${\mathbf{x}}^{\prime}$ means the normalized coordinate and ${\mathbf{\theta^{\prime}}}$ means the normalized design variables; ${{\varvec{\upxi}}}^{\prime}$ indicates the normalized input; ${\mathbb{E}}[\bullet]$ and ${\mathbb{V}}[\bullet]$ indicate the expectation and variance operation along the column vectors.

#### Network structure and loss functions

As described, the input information is a 1-D vector and the output, interested physical quantities, is also a 1-D vector with different size. Thus, the fully-connected (FC) operations are utilized to transform the input feature space to target feature space. For a further description, we use a 1-D vector ${{\varvec{\eta}}}_{i}^{{D}_{i}}\in {\mathbb{R}}^{{D}_{i}}$ with a length of $D_{i}$ to represent the output tensor of *i*-th FC layer. Specially, the input information can be indicated by *i* = 0. Then the mathematic expressions of FC layers can be defined as follows:

18 $$\left\{ {\begin{array}{*{20}c} {{{\varvec{\upeta}}}_{i}^{{D_{i} }} = \sigma ({\mathbf{W}}_{i}^{{D_{{i{ - }1}} ,D_{i} }} {{\varvec{\upeta}}}_{{i{ - }1}}^{{D_{{i{ - }1}} }} + {\mathbf{b}}^{{D_{i} }} );{1} \le i \le N_{l} } \\ {{{\varvec{\upeta}}}_{0}^{{D_{0} }} = {{\varvec{\upxi}}}^{\prime};{{\varvec{\upeta}}}_{{N_{l} }}^{{D_{{N_{l} }} }} = {{\varvec{\uppsi}}}^{\prime}} \\ \end{array} } \right.$$

where $${\mathbf{W}}_{i}^{{D}_{i-1},{D}_{i}}\in {\mathbb{R}}^{{D}_{i-1}\times {D}_{i}}$$ is the weights of *i*-th FC layer with the size of *D*_*i-*1_ × *D*_*i*_ , $${\mathbf{b}}^{{D}_{i}} \in$$$${\mathbb{R}}^{{D}_{i}}$$ is the bias of *i*-th FC layer with size of 1 × *D*_*i*_ and *N*_*l*_ means the number of FC layers. As shown in Eq. (16), a FC layer consists of three operations in sequence: multiply of input vector and weights, the addition of bias and the activation function operation $$\sigma$$ which is used to perform nonlinear transformation. To prevent the problem of vanishing gradient, the activation function ReLU is utilized and the expression can be written as:

19 $${\text{ReLU}}(x) = \max (x,0)$$

It should be noted that the selection of activation function has an important effect on the training and convergence of the deep learning methods. There have been some works on providing more efficient and appropriate activation functions for PINN model. For example, Jagtap et al.^57^ proposed an adaptive activation function with the additional scalable parameter introduced in the network. By leveraging the scalable hyper-parameter, the increased convergence and better performance can be achieved. In the following, they^58^ further extended this scalable parameter from global adjustment to the hidden layer even the neurons, and the results demonstrate the improved training speed and accuracy. Based on the idea of adaptive activation functions, the deep Kronecker neural networks^59^ is proposed by Jagtap. In this paper, we mainly focused on the physical field prediction for the nanofluid in microchannel. Thus, the standard activation function of ReLU is utilized.

In this heat transfer and flow of nanofluids, we employ 10 hidden layers and 32 neurons per hidden layer per output variable (i.e. 4 × 32 = 128 neurons per hidden layer). Since the active function is settled, the learnable parameters $\Theta$ in this network will be the weight matrix and bias vector in each FC layer.

20 $$\Theta = \{ {\mathbf{W}},{\mathbf{b}}\} = \{ {\mathbf{W}}_{i} ,{\mathbf{b}}_{i} |i = 1, \cdots ,N_{l} \}$$

After series transformation of all the FC layers, the direct output is the normalized physical quantities and a reverse normalization operation as shown below is required to get proper predicted physical quantities.

21 $$\{ \hat{p}{,}\hat{u}{,}\hat{v}{,}\hat{t}\}^{T} = {\hat{\varvec{\uppsi}}}{ = }{{\varvec{\uppsi}}}^{\prime} \times \sqrt {{\mathbb{V}}_{{{{\varvec{\uppsi}}} \sim \user2{\mathcal{D}}_{{{\varvec{\uppsi}}}}^{N} }} [{{\varvec{\uppsi}}}]} + {\mathbb{E}}_{{{{\varvec{\uppsi}}} \sim \user2{\mathcal{D}}_{{{\varvec{\uppsi}}}}^{N} }} [{{\varvec{\uppsi}}}]$$

In this reverse normalization method, ${\hat{{\varvec{\uppsi}}}}$ is the predicted physical quantities and ${{\varvec{\uppsi}}}^{\prime}$ is the normalized physical quantities after inverse nondimensionalization obtained at the last layer of the model.

The field reconstruction completed by the deep learning model is a kind of regression method to predict physical quantities from input information. Thus, we utilize a loss function ${\mathcal{L}}_{\psi }$ evaluating the distance between predicted physical quantities and the real ones, which is a loss function of the physical field measurements:

22 $${\mathcal{L}}_{\psi } {(}{{\varvec{\uppsi}}}{,}{\hat{\varvec{\uppsi}}}{;}{\mathbf{W}}{,}{\mathbf{b}}{)} = \sum\nolimits_{i} {\left\| {\psi_{i} { - }\hat{\psi }_{i} } \right\|} { = }\left\| {p{ - }\hat{p}} \right\| + \left\| {u - \hat{u}} \right\| + \left\| {v - \hat{v}} \right\|{ + }\left\| {t - \hat{t}} \right\|$$

For a heat transfer and flow problem, the final simulation results in each control volume should approximately satisfy the conservation laws, including mass, momentum, and energy conservations. To make the most of physical principles, the partial derivatives for physical quantities ${\hat{\varvec{\uppsi}}}{ = }\{ \hat{p},\hat{t},\hat{u},\hat{v}\}^{T}$ are solved by the automatic differential mechanism of PyTorch to acquire the residuals of conservations. The applied residuals of mass, momentum along x and y coordinate and energy conservations are formulated as follows:

23 $$\user2{\mathcal{L}}_{c} {(}{\hat{\varvec{\uppsi}}};{\mathbf{W}}{,}{\mathbf{b}}) = \left\| {\frac{{\partial \hat{u}}}{\partial x} + \frac{{\partial \hat{v}}}{\partial y}} \right\|$$

24 $$\user2{\mathcal{L}}_{mx} {(}{\hat{{\varvec{\uppsi}}}};{\mathbf{W}}{,}{\mathbf{b}}) = \left\| {\hat{u}\frac{{\partial \hat{u}}}{\partial x} + \hat{v}\frac{{\partial \hat{u}}}{\partial y} + \frac{1}{{\rho_{n} }}\frac{{\partial \hat{p}}}{\partial x} - \frac{{\mu_{n} }}{{\rho_{n} }}\left( {\frac{{\partial^{2} \hat{u}}}{{\partial x^{2} }} + \frac{{\partial^{2} \hat{u}}}{{\partial y^{2} }}} \right)} \right\|$$

25 $$\user2{\mathcal{L}}_{my} {(}{\hat{\varvec{\uppsi}}};{\mathbf{W}}{,}{\mathbf{b}}) = \left\| {\hat{u}\frac{{\partial \hat{v}}}{\partial x} + \hat{v}\frac{{\partial \hat{v}}}{\partial y} + \frac{1}{{\rho_{n} }}\frac{{\partial \hat{p}}}{\partial y} - \frac{{\mu_{n} }}{{\rho_{n} }}\left( {\frac{{\partial^{2} \hat{v}}}{{\partial x^{2} }} + \frac{{\partial^{2} \hat{v}}}{{\partial y^{2} }}} \right)} \right\|$$

26 $$\user2{\mathcal{L}}_{e} {(}{\hat{\varvec{\uppsi}}};{\mathbf{W}}{,}{\mathbf{b}}) = \left\| {\hat{u}\frac{{\partial \hat{t}}}{\partial x} + \hat{v}\frac{{\partial \hat{t}}}{\partial y} - \alpha_{n} \left( {\frac{{\partial^{2} \hat{t}}}{{\partial x^{2} }} + \frac{{\partial^{2} \hat{t}}}{{\partial y^{2} }}} \right)} \right\|$$

where $\user2{\mathcal{L}}_{c}$, $\user2{\mathcal{L}}_{mx}$, $\user2{\mathcal{L}}_{my}$ and $\user2{\mathcal{L}}_{e}$ means the loss function of mass, momentum along *x* coordinate, momentum along *y* coordinate, and energy conservations, respectively, and $\widehat{p}$, $\widehat{t}$, $\widehat{u}$ and $\widehat{v}$ denotes the predicted physical quantities. The more accurate the predicted physical fields $\widehat{{\varvec{\psi}}}$, the closer to zero the loss function of conservation laws $\user2{\mathcal{L}}_{g} = \{ \user2{\mathcal{L}}_{c} ,\user2{\mathcal{L}}_{mx} ,\user2{\mathcal{L}}_{my} ,\user2{\mathcal{L}}_{e} \}$ is, which means that the control volumes approximately respect the conservation laws. The total loss function is composed by the field loss function and a weighted sum of conservation loss functions:

27 $$\user2{\mathcal{L}}_{total} {(}{\hat{\varvec{\uppsi}}}{,}{{\varvec{\uppsi}}};{\mathbf{W}}{,}{\mathbf{b}}{) = }\user2{\mathcal{L}}_{\psi } + \lambda_{\user2{\mathcal{L}}} [\user2{\mathcal{L}}_{c} + \user2{\mathcal{L}}_{mx} + \user2{\mathcal{L}}_{my} + \user2{\mathcal{L}}_{e} ]$$

The weight of conservations $\lambda_{\user2{\mathcal{L}}}$ aims at balancing the scale of field loss and conservation loss. The training is a search process for a pair of optimal weight matrix ${\mathbf{W}}^{*}$ and bias ${\mathbf{b}}^{*}$ to minimize the total loss function. As shown in Eq. (28), the optimal weight matrix ${\mathbf{W}}^{*}$ and bias ${\mathbf{b}}^{*}$ are the hyperparameters when total loss function $\user2{\mathcal{L}}_{total}$ reaches minimized value. It should be emphasized that this work focuses on the improvement of the incorporation with conservation laws, thus the boundary condition and initial conditions are removed from loss functions. The investigation of boundary and initial conditions can be found in the application of PINN on cardiovascular flows^49^.

28 $$\varvec{\ominus }{{\{ }}{\mathbf{W}}^{*},{\mathbf{b}}^{*}{{\} }} = \mathop {{\text{argmin}}}\limits_{{\{ {\mathbf{W}},{\mathbf{b}}\} }} \, \varvec{\mathcal{L}}_{total} ({\hat{\varvec{\uppsi}}},{{\varvec{\uppsi}}};{\mathbf{W}},{\mathbf{b}})$$

#### Performance criteria

Since the deep learning model can predict physical quantities for one sample point in one set, the whole physical fields of a case can be reconstructed by all corresponding sample points fed to model. For better testing and verifying the model, we evaluate the reconstruction power with four criteria in whole fields.

Two field error criteria named relative *L*_1_ and relative *L*_2_ for each field are used as a metric for evaluation^45^. The calculation for them are presented as follows:

29 $$\left\{ {\begin{array}{*{20}c} {L_{1} (\psi ,\hat{\psi }) = {{\sum\limits_{i = 1}^{N} {\left\| {\psi^{i} - \hat{\psi }^{i} } \right\|_{1} } } \mathord{\left/ {\vphantom {{\sum\limits_{i = 1}^{N} {\left\| {\psi^{i} - \hat{\psi }^{i} } \right\|_{1} } } {\sum\limits_{i = 1}^{N} {\left\| {\psi^{i} } \right\|_{1} } }}} \right. \kern-\nulldelimiterspace} {\sum\limits_{i = 1}^{N} {\left\| {\psi^{i} } \right\|_{1} } }}} \\ {L_{2} (\psi ,\hat{\psi }) = {{\sum\limits_{i = 1}^{N} {\left\| {\psi^{i} - \hat{\psi }^{i} } \right\|_{2} } } \mathord{\left/ {\vphantom {{\sum\limits_{i = 1}^{N} {\left\| {\psi^{i} - \hat{\psi }^{i} } \right\|_{2} } } {\sum\limits_{i = 1}^{N} {\left\| {\psi^{i} } \right\|_{2} } }}} \right. \kern-\nulldelimiterspace} {\sum\limits_{i = 1}^{N} {\left\| {\psi^{i} } \right\|_{2} } }}} \\ \end{array} } \right.$$

where ${\Vert \cdot \Vert }_{1}$ means the norm-1 operation, ${\Vert \cdot \Vert }_{2}$ means the norm-2 operation, and *N* is the number of sample points of the field in one case. Except for those two normal criteria indicating the deviations of predicted quantities, we also adopt the residuals of governing equations (mass conservation R_c_, *x* momentum conservation R_mx_, *y* momentum conservation R_my_ and energy conservation R_e_) referencing from the numerical software FLUENT (Eq. (28–5) in chapter 28.15 of Fluent help document) to evaluate the accuracy of physical fields.

In addition, two common performance characteristics, Nusselt number *Nu* and fanning friction factor *f* to test the accuracy of predicted fields. The *Nu* representing thermal performance is calculated as:

30 $$Nu = \frac{{hD_{h} }}{{\lambda_{{\text{n}}} }}{ = }\frac{q}{{\overline{t}_{{\text{w}}} - \overline{t}_{{\text{f}}} }}\frac{2H}{{\lambda_{{\text{n}}} }}$$

where *h* is heat transfer coefficient; $q$ is heat flux density which is a constant for each case; $\overline{t}_{w}$ and $\overline{t}_{f}$ denote the averaged wall temperature and averaged bulk temperature. The *f* indicating the hydraulic performance is calculated as:

31 $$f = \frac{{\Delta \overline{p}D_{h} }}{{2\rho_{{\text{n}}} \Delta a\overline{u}_{{{\text{in}}}}^{2} }} = \frac{{(\overline{p}_{{{\text{in}}}} - \overline{p}_{{{\text{out}}}} )H}}{{\rho_{{\text{n}}} (a - {2}a_{1} )\overline{u}_{{{\text{in}}}}^{2} }}$$

where $t_{w}$ is the mean temperature of top and bottom walls; $t_{f}$ is mean fluid temperature; $p_{{{\text{in}}}}$ is the average pressure at inlet; and $p_{{{\text{out}}}}$ is the average pressure at outlet. They are shown in Eq. (34).

32 $$\left\{ {\begin{array}{*{20}c} {\overline{p}_{{{\text{in}}}} = \int_{{{\text{in}}}} {p(x,y)/ady} } \\ {\overline{p}_{{{\text{out}}}} = \int_{{{\text{out}}}} {p(x,y)/ady} } \\ {\overline{t}_{f} = \iint_{{_{{{\text{fluid}}}} }} {t(x,y)/V_{{{\text{fluid}}}} dxdy}} \\ {\overline{t}_{w} = \frac{{\int_{top} {t(x,y)dl} + \int_{bottom} {t(x,y)dl} }}{{L_{top} + L_{bottom} }}} \\ \end{array} } \right.$$

The relative error (RE) for performance characteristics *Nu* or *f* can be calculated as:

33 $${\text{RE}} = \frac{{|\hat{y} - y|}}{y} \times 100\%$$

where $y$ is the parameter calculated from original fields and $\hat{y}$ is the predicted value extracted from the reconstructed fields. On some level, *Nu* and *f* criteria can be considered as another integral weighted form of the field error.

## Results

### Performance analysis of reconstruction

In this section, we demonstrate that the improved model can reliably reconstruct physical fields for nanofluids flowing in microchannels. The training dataset consists of 3000 unique cases (3000 × 550 × 40 sample points) randomly selected from 6000 cases in total. And the remaining 3000 cases are used as test dataset to illustrate the reconstruction performance from four aspects: physical field visualizations, physical field error, residuals of conservation equations and relative error of performance characteristics.

**Figure 3:**
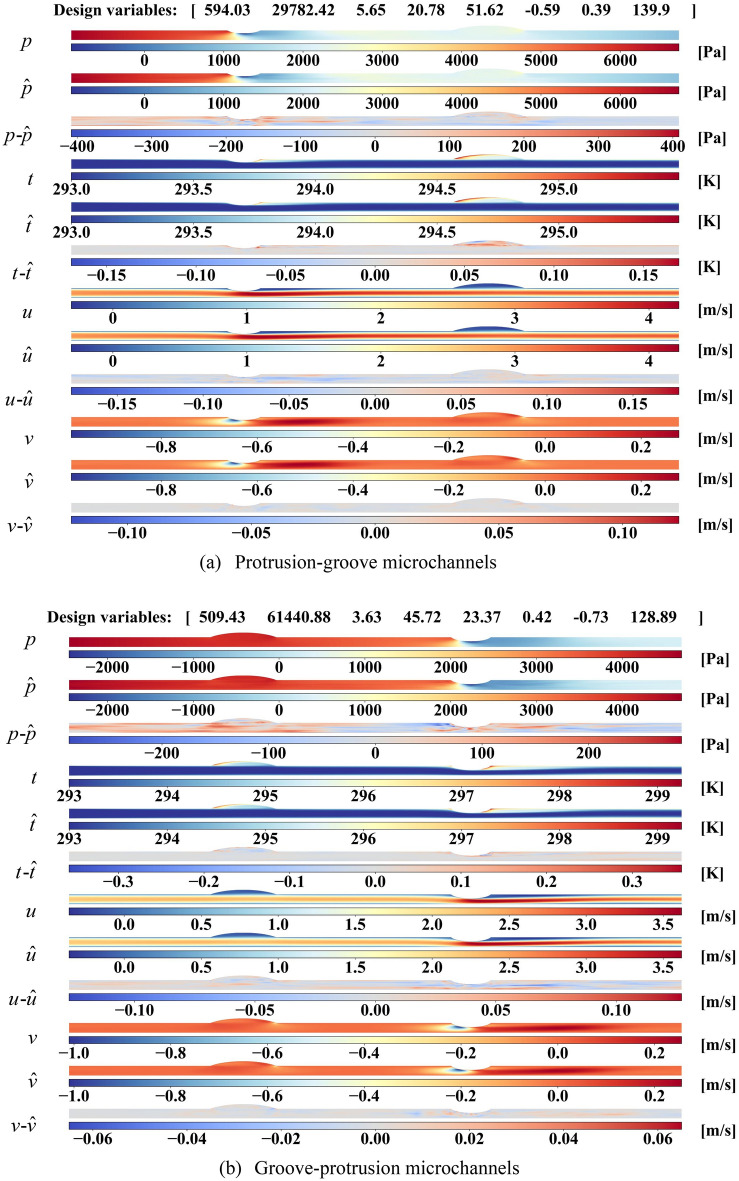

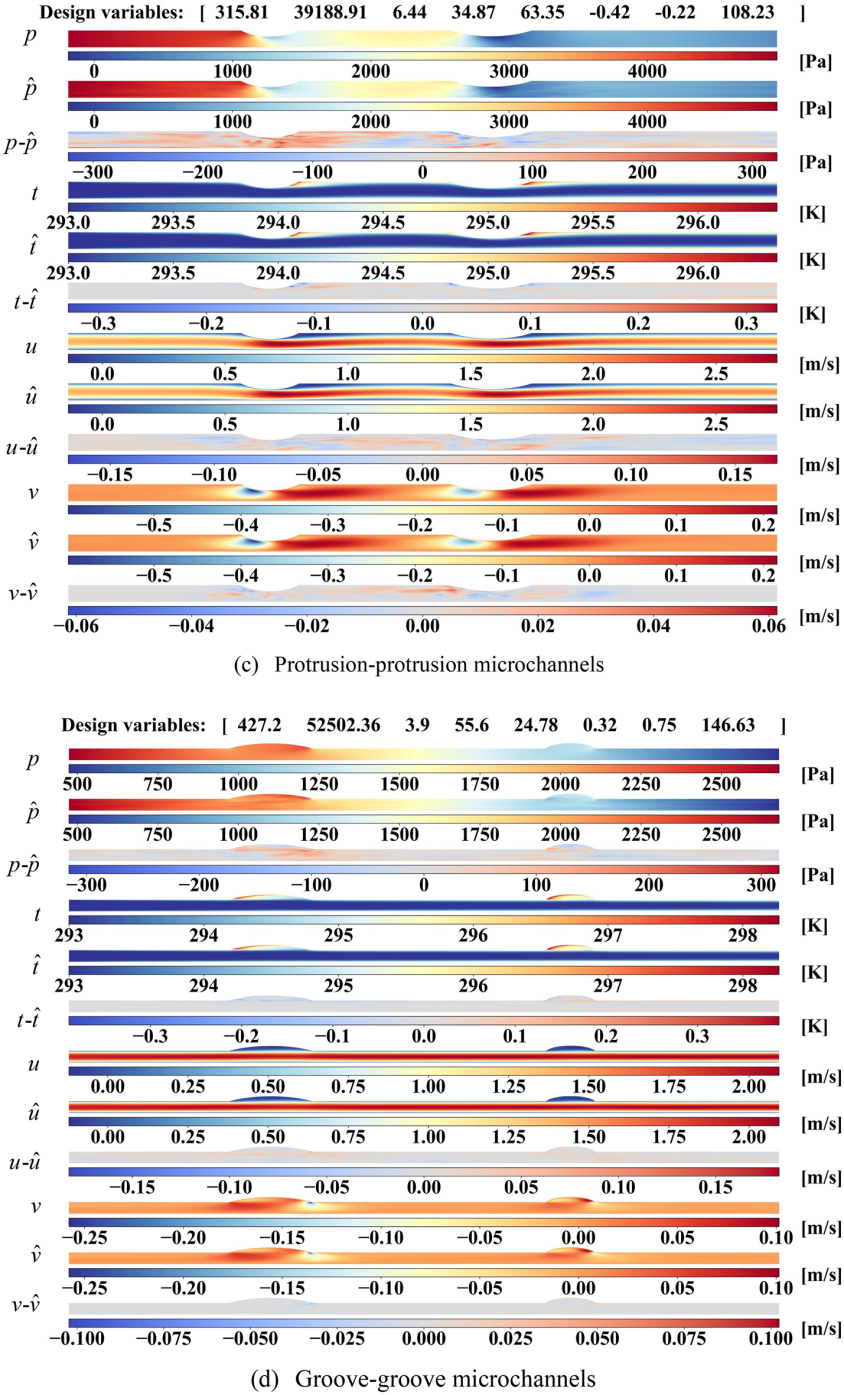
Flow field reconstructions with different geometry (two specific examples for each geometric type of microchannels) and the corresponding ground truth.

A collection of examples with varying design parameters is presented to visualize the true, predicted physical fields and absolute error distributions in Fig. 3. Based on the geometry, the microchannels can be divided into four types: protrusion-groove, groove-protrusion, protrusion-protrusion and groove-groove microchannels. Considering the different flow and heat transfer behaviors for four types of microchannels, two examples for each type of microchannels are displayed. Besides, the design variables ($[Re,q, \varphi ,R_{1} ,R{}_{2},\delta_{1} ,\delta_{2} , a_{3} ]$) are listed at the top of each image to describe the corresponding case.

It can be seen from Fig. 3 that true and predicted pressure both drop down along microchannel and reduces to zero at the outlet as nanofluids developing. Compared with smooth pressure fields of ground truth, the model can reconstruct the pressure field identically in a coarse structure with few discontinuous lines in dramatically changing parts. Due to the heat flux conditions on walls, the nanofluids near walls is heated along microchannel while the temperature of nanofluids in middle keeps around the inlet temperature of 293 K. It should be noted that the two obvious high-temperature zones are found in the leading edge of groove or the trailing edge of protrusion, respectively. Even with some dramatic changes in temperature distributions, our approach can generate plausible temperature fields that close to ground truth. And the prediction error mainly concentrated around dramatical changes areas, especially for the leading and trailing edge of groove/protrusion. As for velocity *u*, different phenomena are observed around grooves and protrusions. In protrusion, a high-velocity zone forms when the microchannel narrows and then flows towards the top wall after the groove. No significant change is found in groove except for lower *u* in the expansion of microchannel. From the plots in Fig. 3, the reconstructed *u* distributions are consistent with the true distributions. The distribution of velocity *v* is much more complex, which a low-velocity zone and a high-velocity zone are formed around grooves and protrusions. Though it can be found that the absolute error is much large compared with the velocity field, the large errors only exist at local points, making little influence on global velocity distributions. It can be inferred that predicted velocity *v* fields resemble the ground truth flow fields. As discussed above, our model can predict plausible physical fields, including pressure, temperature, and velocities, for all kinds of microchannels.

**Figure 4:**
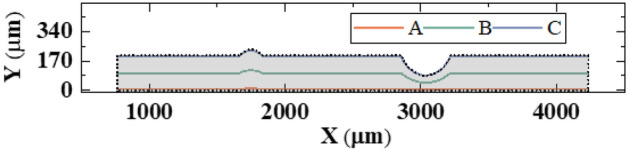
The three curves A, B and C in microchannel.

**Figure 5:**
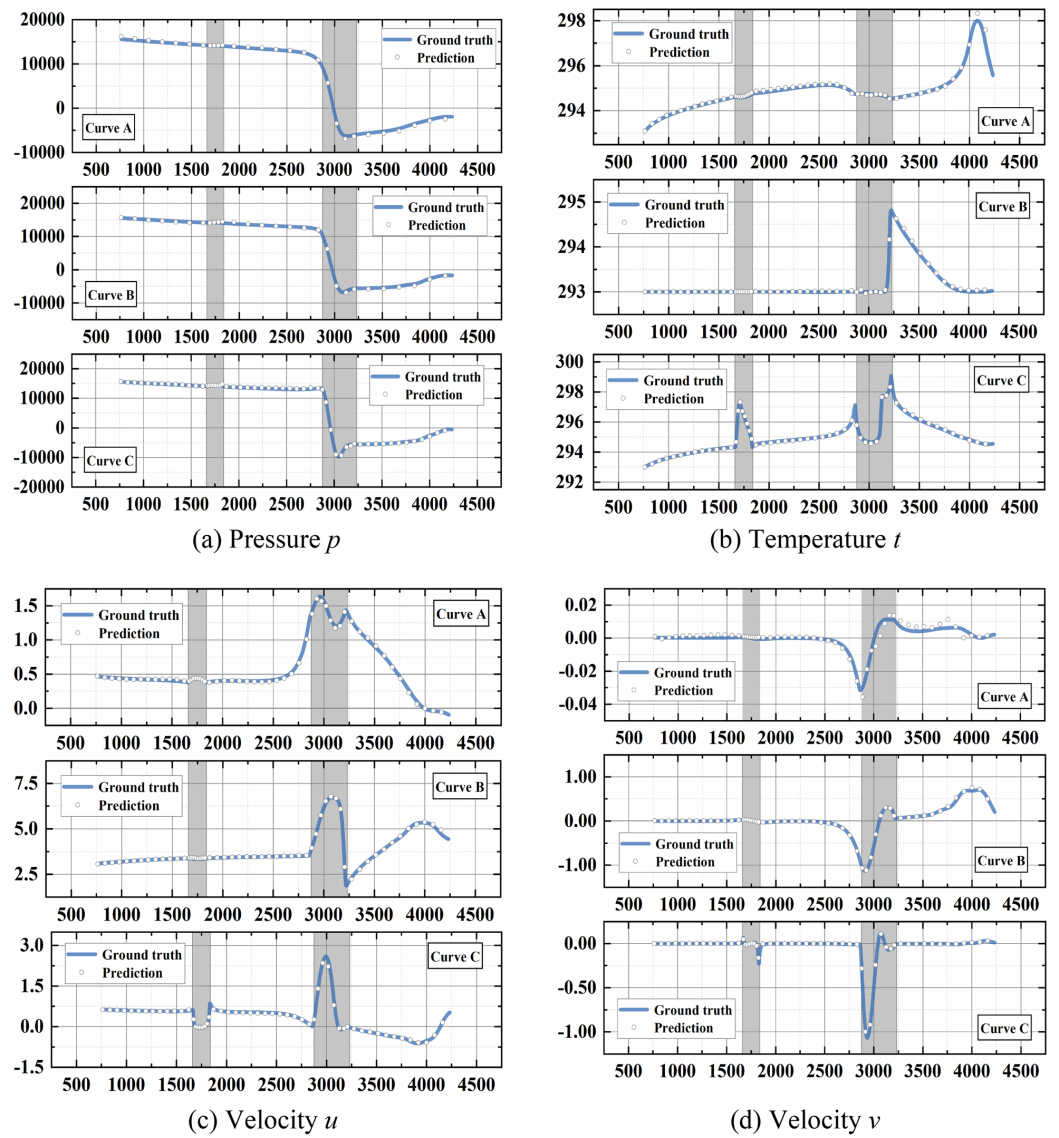
The detailed distributions along curve A, B and C.

In Fig. 4, three curves A, B and C are plotted in microchannels to describe the prediction performance near bottom wall, the center of microchannel and near top wall, respectively. Along three curves, the comparisons between predicted pressure, temperature and velocity (*u*, *v*) attributes and true fields are presented in Fig. 5, where the shaded areas indicate the groove and protrusion. As shown in plots, the model can favorably predict four field attributes in detail, while velocity *v* is found close to ground truth with marginal deviations in curve A. This may be partly due to the low values of velocity *v* near wall. This brilliant prediction ability enables the deep learning model to gather arbitrary points of physical attributes with favorable accuracy and analyze the heat transfer and flow behavior.

**Figure 6:**
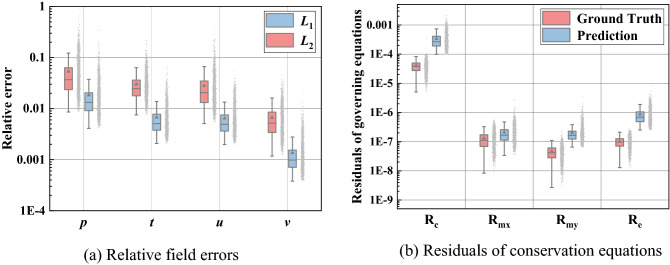
Prediction performance of relative field error and conservation equations.

To quantitatively investigate the reconstruction performance, the two evaluation criteria relative *L*_1_ and *L*_2_ error as well as the residuals of four conservation equations, mass, momentum, and energy, are discussed in Fig. 6. For four physical attributes, all averaged *L*_1_ and *L*_2_ error is lower than 0.02 and 0.1. The low field criteria distributions indicate that our approach can reconstruct fields for different design variables with high fidelity. From Fig. 6b, the residuals of predicted conservation equations are close to the true residuals and the maximum deviation may be the residual of predicted mass equation which is tenfold true residual. This result illustrates that the reconstructed physical fields almost satisfy the conservation equations. In some way, it can be conjectured that the physical-informed model no longer simply reconstructed physical fields, but approximate the inherent physical laws based on prior information of conservation equations to improve the reconstruction performance.

**Figure 7:**
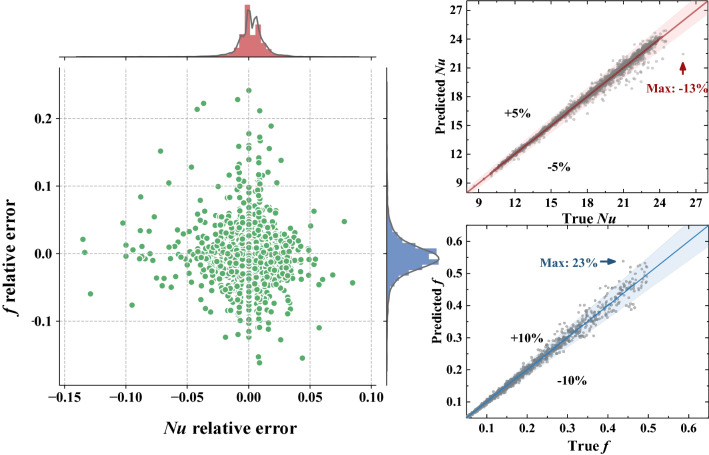
Prediction of thermal and hydraulic performance.

In flow and heat transfer problem, the most important performance characteristics are Nusselt number *Nu* representing thermal performance and Fanning friction factor *f* representing hydraulic performance. In recent decades, numerous studies focused on the surrogate models predicting *Nu* and *f* based on measurements or design variables. Herein, the surrogate model is replaced by the reconstruction model, and the characteristics can be extracted from generated fields by integral operations shown in Eq. (32). In Fig. 7, we present the characteristics prediction performance. It is observed that the relative errors of two performance characteristics distributed around 0 with negligible bias. Besides, the relative error of *Nu* is high to -13% while it is less than 5% for most examples. Likewise, though the maximum relative error of *f* is high to 23%, most errors are less than 10%. It can be inferred that the accuracies of performance characteristics are high enough to complete diverse engineering applications in the range of studied cases. Overall, the proposed physics-informed reconstruction framework generalizes well for the tasks of field reconstruction and performance prediction.

### Compared with classical surrogate models

In the traditional design process, numerical simulations are conducted over and over upon the previous unqualified designs until a satisfying result is obtained. Although the computational ability is powerful nowadays, the design periodic is still dragged by massive numerical simulations which is time-consuming for iterative solution of high-dimensional equations. To accelerate the design process, numerical simulations are replaced by surrogate models to approximate the objective functions, such as *Nu* and *f*. However, single surrogate model can only construct one mapping function, and the surrogate model should be renewed once the objective functions changed. Nearly all the objective function is a kind of abstract for physical fields which means that they can be extracted from fields by mathematical methods. Based on this fact, our approach obtains the objective functions directly from physical fields predicted by PINNs, which makes it possible to get any multiple mapping functions together.

**Figure 8:**
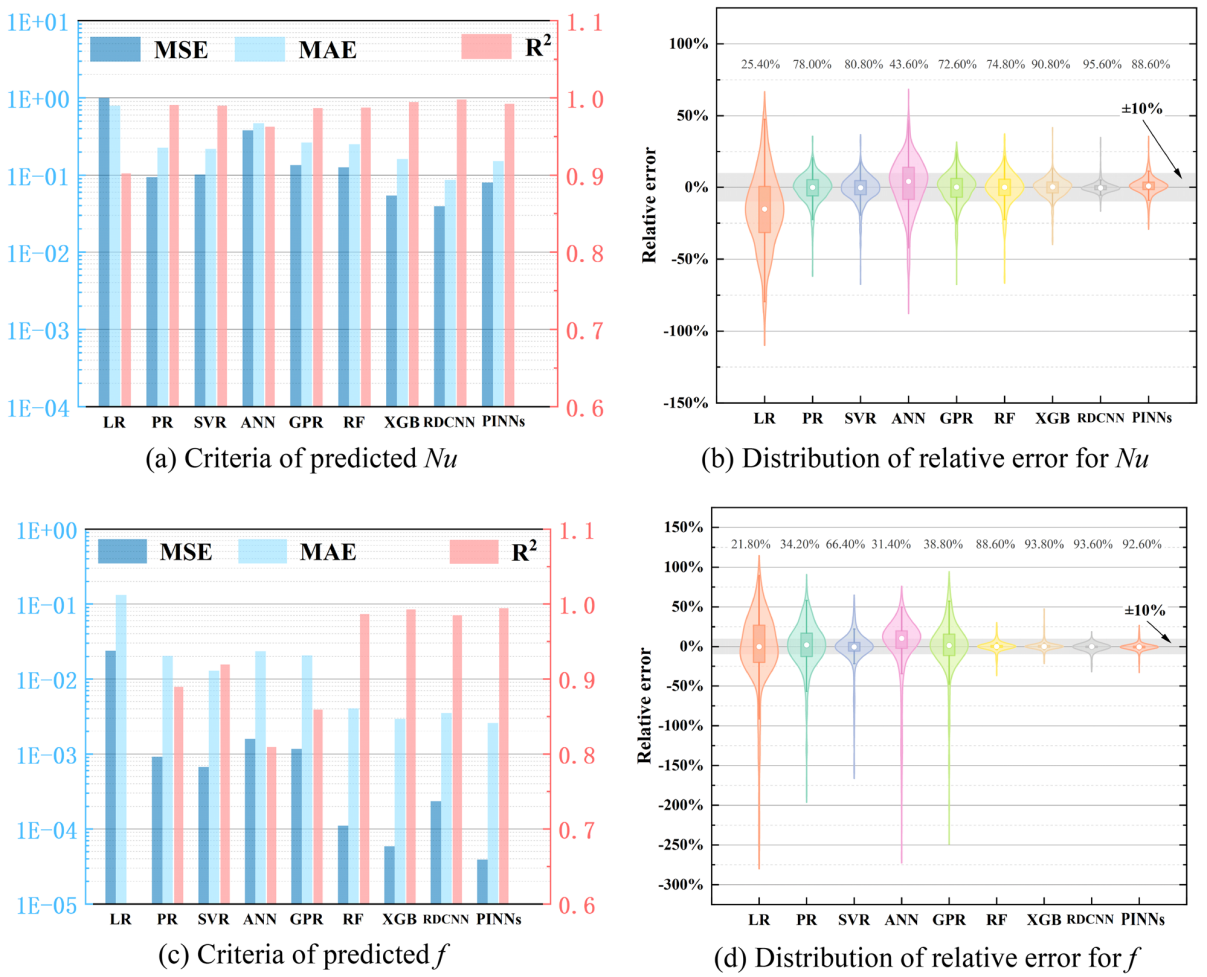
Comparison of prediction performance between different models.

In flow and heat transfer problems, performance characteristics *Nu* and *f* are widely used as objective functions. Thus, the prediction performance of different traditional surrogate models including Linear Regression (LR), Polynomial Regression (PR), Supported Vector Regression (SVR), Artificial Neural Network (ANN), Gauss Process Regression (GPR), Random Forest (RF), Extreme Gradient Boosting (XGB), our previous reconstruction model constructed by the deep convolutional neural network^36^ (RDCNN) and our approach in this paper for *Nu* and *f* are plotted in Fig. 8. It should be noted that RDCNN and PINNs are both reconstruction models and they adopt same extraction method of *Nu* and *f* from physical fields. However, the RDCNN is different from PINNs by its image-treated physical fields and deep convolutional neural network for reconstruction. To distinguish the prediction performance, three global criteria R-square (R^2^), mean squared error (MSE) and mean absolute error (MAE) and relative error (RE) of performance characteristics are utilized. The mathematical expressions of three global criteria are described below:

34 $$\left\{ {\begin{array}{*{20}c} {{\text{MAE}} = \frac{1}{N}\sum\limits_{i = 1}^{N} {{|}y - y^{\prime}{|}} } \\ {{\text{MSE}} = \frac{1}{N}\sum\limits_{i = 1}^{N} {(y - y^{\prime})^{2} } } \\ {{\text{R}}^{{2}} = 1 - {{\sum\limits_{i = 1}^{N} {(y - y^{\prime})^{2} } } \mathord{\left/ {\vphantom {{\sum\limits_{i = 1}^{N} {(y - y^{\prime})^{2} } } {\sum\limits_{i = 1}^{N} {(y - \overline{y})^{2} } }}} \right. \kern-\nulldelimiterspace} {\sum\limits_{i = 1}^{N} {(y - \overline{y})^{2} } }}} \\ \end{array} } \right.$$

where *y* means the original performance characteristics, $y^{\prime}$ means the performance characteristics predicted by surrogate models, $\overline{y }$ is the mean value of original performance characteristics and *N* is the total number of testing dataset, which is 3000 for this comparison (3000 samples are applied as training dataset).

It can be observed from Fig. 8a,b that reconstruction model RDCNN shows the best prediction performance of *Nu* ,and our model is slightly lower than RDCNN with 0.992 R^2^ score, 0.081 MSE, 0.152 MAE and 88.6% for ± 10% Relative Error (RE), respectively. Among other classical surrogate models, XGB provides best prediction accuracy which is still lower than our reconstruction models. For the prediction performance of *f*, the improved model outperforms other models with 0.994 R^2^ score, 3.89 × 10^–5^ MSE, 2.58 × 10^–4^ MAE and 92.6% for ± 10% Relative Error (RE). As for other classical surrogate models, the prediction power of XGB is far more than others, but it is still inferior to reconstruction models considering comprehensive performance. The results confirm that the reconstruction models are beneficial to predict performance characteristics based on predicted physical fields compared with regular surrogate models.

### Effect of training size

An inherent problem of data-driven machine learning approaches is that model performance strongly depends on the quality of training dataset. Thus, it is significant to investigate the effect of training size on the reconstruction performance of our reconstruction model. In this section, four training sizes are utilized: 3000 groups of sample points (50%), 2000 groups (33%), 1000 groups (17%) and 500 groups (8%) for all 6000 groups in raw data. Besides, the remain 3000 groups of sample points are taken as testing dataset for all the models with different training size. In the following, the effect of training size is studied from three factors: relative errors of physical fields, residuals of conservation equations and relative errors of performance characteristics.

**Figure 9:**
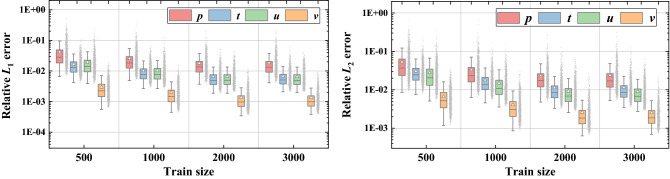
Relative *L*_1_ and *L*_2_ errors vs. Train size.

Figure 9 shows the comparisons of relative field errors *L*_1_ and *L*_2_ for four different training size. It is apparent that physical relative errors decrease as training size enlarges from 500 to 3000 groups of sample points and the decrement drops down gradually. Averaged physical errors *L*_1_ and *L*_2_ for all physical fields are under 0.05 with training size greater than 2000 (33%). Once the training size is up to 2000, increasing training size shows a very limited improvement of reconstruction accuracy.

**Figure 10:**
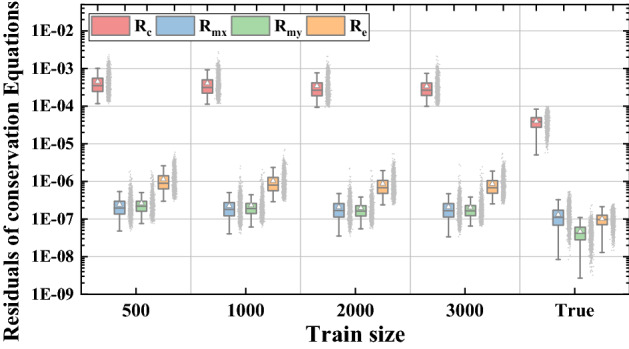
Residuals of conservation equations vs. train size.

In Fig. 10, it is observed that the residuals of predicted conservation equations with four training size are all below 2 × 10^–3^, while the residuals of true conservation equations are lower than 10^–4^. Similar to the physical field relative errors, the residuals of conservation equations keep decreasing till training size is higher than 2000. The varying trend indicates that the reconstruction performance is difficult to be improved through reducing residuals of conservation equations with adequate training samples (2000 groups of sample points in this study).

**Figure 11:**
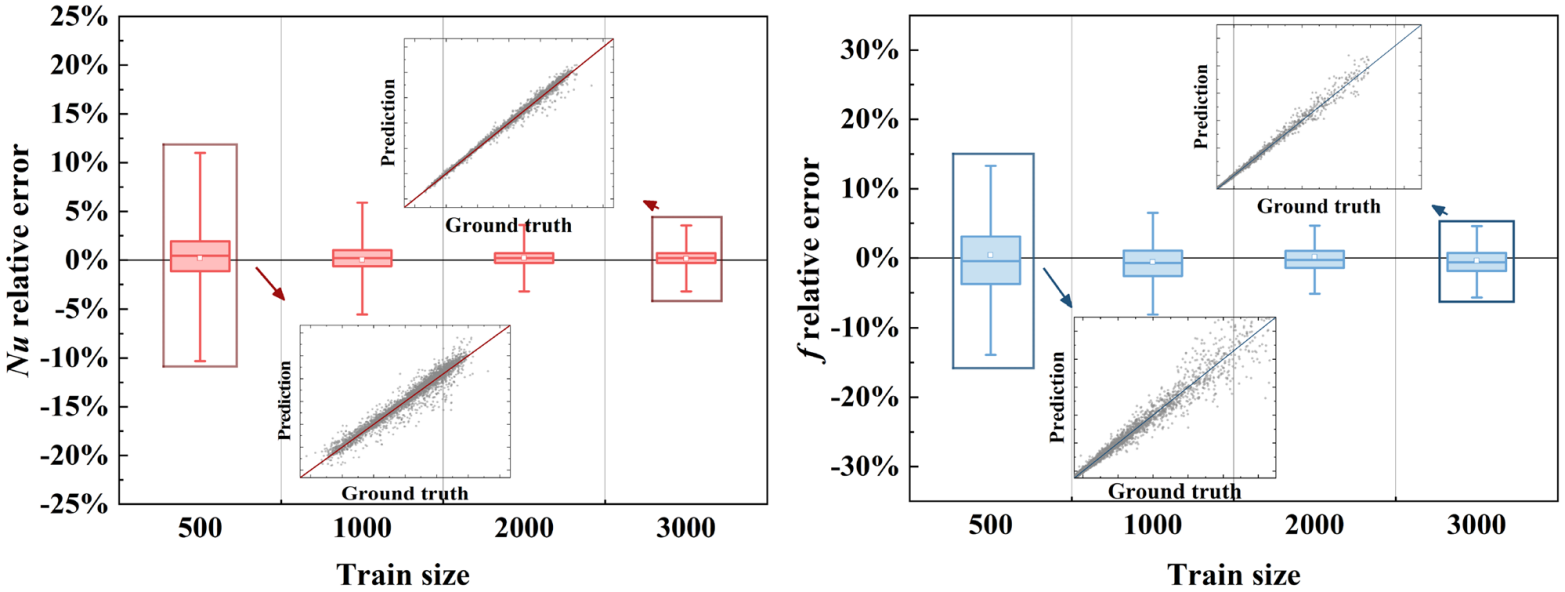
Relative errors of performance vs. Train size.

In addition, the relative errors of attention-attracting performance characteristics *Nu* and *f* are presented in Fig. 11. From the box plots for relative error of *Nu*, it can be found that the relative error of thermal performance is distributed around 0 and the maximum relative error decreases from 12 to 5% with training size increasing from 500 to 3000 groups. As for hydraulic performance *f*, the averaged relative error fluctuates around 0 and the maximum relative error also decreases from 15 to 8% with larger training size. The higher relative error for *f* may due to the much smaller values of *f* and the higher relative errors of pressure attribute. Similar to physical fields relative errors and residuals of conservation equations, the relative errors of performance characteristics show little reduction when training size is larger than 2000.

Overall, the increase of training size has an obvious positive effect on reconstruction performance, including reducing physical fields relative errors, regulating residuals of conservation equations and decreasing relative errors of performance characteristics. However, this improvement of reconstruction power is constrained if the training samples are adequate. In the light of this study, training data of 2000 groups is enough to attain excellent PINN model with acceptable accuracy.

### Extrapolation performance

As discussed above, the applied physics-informed model shows excellent capability for multiple evaluation criteria of interested fields and characteristics. However, it should be noted that the reconstruction of fields above can be regarded as interpolation due to randomly divided datasets whose design variables of training and testing set are under the same probability distribution. To demonstrate the generality and scalability of our method, it is an intuitive idea to investigate extrapolation power of the approach—one of the most important problem but perhaps rarely mentioned in recent research. In this section, we evaluate the extrapolation capabilities from two aspects: physical fields relative errors *L*_2_ and relative errors of performance characteristics, *Nu* and *f*. Moreover, we repartition different training data set based on the extrapolation of four important parameters *Re*, *q*, *φ* and *a*_3_, covering the design information related to geometry, nanofluids and boundary conditions.

**Figure 12:**
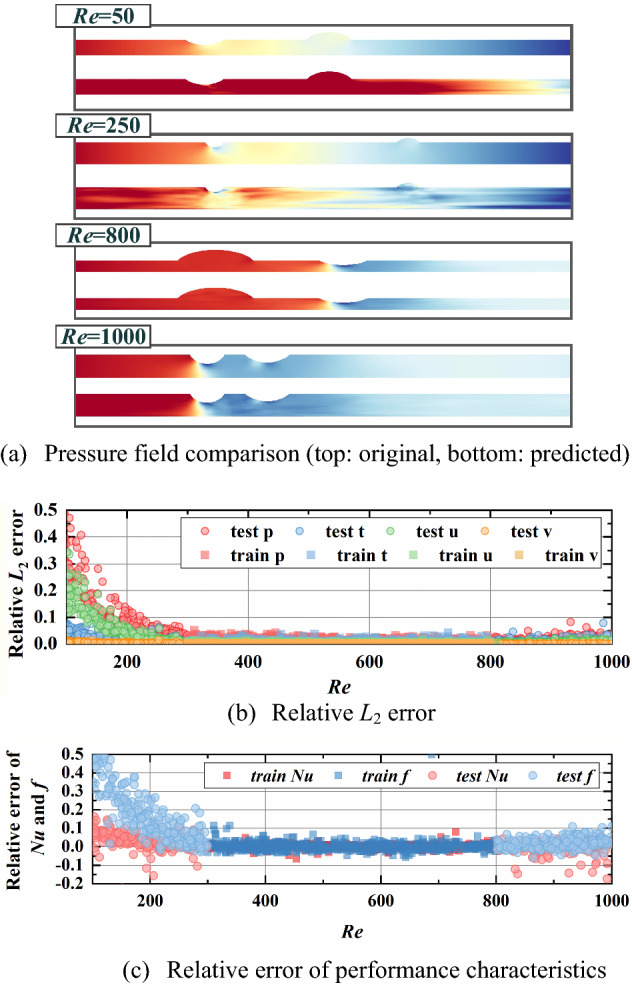
Extrapolation performance of *Re.*

**Figure 13:**
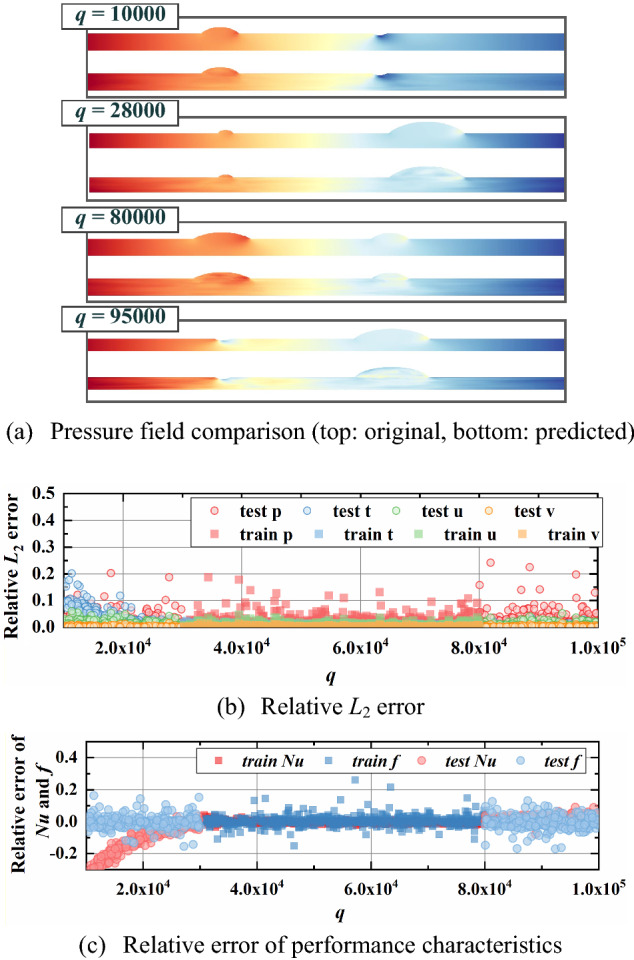
Extrapolation performance of *q.*

**Figure 14:**
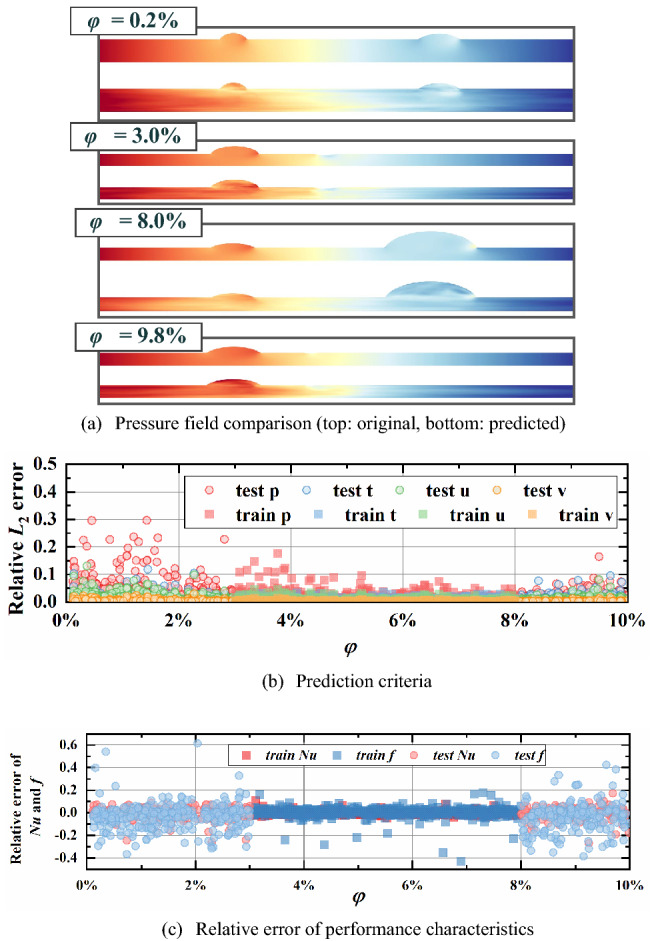
Extrapolation performance of *φ.*

**Figure 15:**
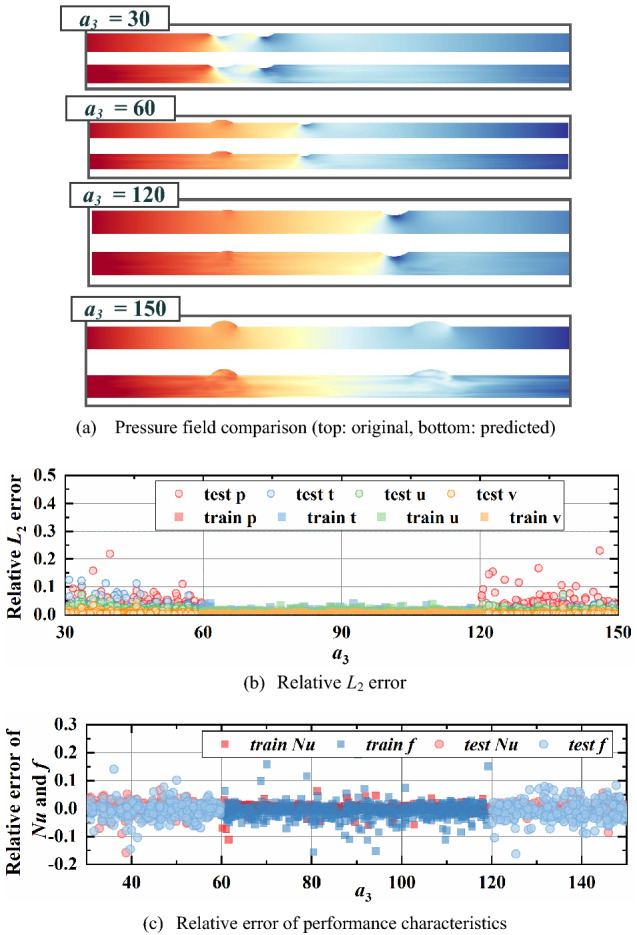
Extrapolation performance of *a*_3_.

From Figs. 12, 13, 14, 15, the relative *L*_2_ error of physical fields and relative error of characteristics are plotted with varying design variables for training and testing datasets. In addition, the pressure fields in the testing dataset are displayed for visual observation. An obvious result can be found that relative *L*_2_ error and relative error of performance rise gradually as design variables extend outward the training ranges. This indicates a noticeable conclusion that the farther the design variables are away from the training interval, the worse the reconstruction performance. The design variable *a*_3_, which provides the least impact on the physical fields, has the best extrapolation performance. It is observed that the reconstructed fields in testing dataset show a relatively good match with the ground truth, the relative error *L*_2_ is less than 0.1 except some special cases and the relative error of characteristics concentrated between -10% and 10%. Then the extrapolation performance of *φ* ranks second, with reasonable physical fields, slightly higher relative error *L*_2_ and larger relative error of performance.

As for the boundary conditions of *Re* and *q*, the extrapolation performance along increasing variables is pretty good while a clear accelerating downward trend of relative *L*_2_ error and an increasing bias of increasing relative error of performance can be observed with decreasing variable. For the extrapolation of *Re*, the relative *L*_2_ errors of pressure and velocity *u* up to 0.5 and the relative error of *f* ups to 50% with lower *Re* while the relative error of *Nu* is much smaller. Besides, the reconstructed pressure fields can only obtain coarse flow patterns in low *Re* while plausible predictions of pressure fields are found with high *Re*. The reason for this result is the significant influence of varying *Re* on pressure. Likewise, due to the close relationship between *q* and temperature field, the relative *L*_2_ errors of temperature field (maximum of 0.5) and the relative error of *Nu* (maximum of 25%) is much higher with decreasing *q*. Besides, the reconstructed physical fields agree well with ground truth with lower *q*. The results show that our model can predict multiple physical fields and performance characteristics with favorable accuracy if *Re* and *q* increase, and acceptable accuracy if *Re* and *q* slightly decrease.

In summary, the prediction performance become worse with design variables deviated from training ranges and this is determined by the regression essence of neural network. Besides, the larger the influence of design variable on fields, the less satisfactory the extrapolation performance. Even though the accuracy decreases with the design variable extending, our approach can reconstruct plausible physical fields and predict the thermal and hydraulic performance accurately. Especially for φ and a3, the relative error of performance characteristics ranging from -10% to 10% for almost all cases and the physical fields visualizations are in good agreement with ground truths. It indicates that our model enables relatively accurate prediction out of the training range to some extent.

## Conclusion

In this study, a physics informed deep neural network incorporating the first principle, which is conservation laws in thermal and fluids mechanism, is proposed to reconstruct physical fields for nanofluids convection with design variables as input, including nanofluids volume fraction, geometric parameters and boundary conditions parameters. The main results are concluded as follows:

1. The prediction power of our model is validated from four factors: the physical fields visualizations resemble the ground truth with reasonable details; the relative *L*_1_ and *L*_2_ for physical fields are quite lower than 0.02 and 0.1; the residuals of conservations close to the results of numerical simulations and the relative error for *Nu* and *f* are less than 10% for most cases.
2. Compared with classical surrogate models, reconstruction models show superior prediction performance of either *Nu* or *f* due to reconstructed fields (RDCNN ranks 1 for *Nu* and our model ranks 1 for *f*).
3. As indicated in the results with different training sizes, the more sample points involved in training, the more powerful the physics-informed model is for reconstructing physical fields. In nanofluids convection, 2000 groups of sample points enable the physics-informed model to achieve best prediction performance approximately.
4. The evaluations of reconstruction performance with extended design variables demonstrate that the proposed model shows certain parametric extrapolation ability for the heat transfer and flow of nanofluids.

## List of symbols

${\varvec{\psi}}$: True physical quantities
$\widehat{{\varvec{\psi}}}$: Predicted physical quantities
R: Conservation residual
$${\mathbf{x}}$$: Coordinates vector
${\mathbf{x}}^{\prime}$: Coordinate after normalization and nondimensionalization
*x*: X coordinate (μm)
*y*: Y coordinate (μm)
*x*^***^: Non-dimensional *x* coordinate
*y*^***^: Non-dimensional *y* coordinate
*b*_*f*_: Body force
**u**: Velocity vector
*p*: Pressure (Pa)
*t*: Temperature (K)
*u*: Velocity along *x* coordinate (m/s)
*v*: Velocity along *y* coordinate (m/s)
*p*^***^: Non-dimensional pressure
*t*^***^: Non-dimensional temperature
*u*^***^: Non-dimensional x velocity
*v*^***^: Non-dimensional y velocity
$\widehat{p}$: Predicted pressure (Pa)
$$\widehat{t}$$: Predicted temperature (K)
$$\widehat{u}$$: Predicted x velocity (m/s)
$$\hat{v}$$: Predicted y velocity (m/s)
*H*: Microchannel height (μm)
*d*: Groove depth (μm)
*a*: Length of microchannel
*a*_1_: Length of extension (μm)
*a*_2_: Length between inlet and first groove/protrusion (μm)
*a*_3_: Groove interval (μm)
$$u_{\infty }$$: Inlet velocity (m·s^−^^1^)
$C_{P}$: Specific heat (J·kg^−^^1^·K^−^^1^)
$q$: Heat flux of boundary (W·m^−^^2^)
*R*: Groove radius (μm)
*D*_h_: Hydraulic length (μm)
*D*_**θ**_: The dimensions of variable parameters
*P**r*: Prandtl number
*Nu*: Nusselt number
*f*: Fanning friction factor
*Re*: Reynolds number
*t*_f_: Mean temperature of fluid (K)
*t*_w_: Mean temperature of walls (K)
Δ*t*: Temperature difference (Pa)
Δ*p*: Pressure drop along channel (Pa)
*N*_s_: Number of sample points in training dataset
${\mathbb{F}}\left(\bullet \right)$: Traditional numerical or experimental methods
${\tilde{\mathbb{F}}}\left(\bullet \right)$: Approximate mapping for physical fields
**b**^*^: The optimal Bias of fully-connected layer
$${\mathbb{E}}[\bullet]$$: Expectation operation
$${\mathbb{V}}[\bullet]$$: Variance operation
${\mathbf{W}}$: Weights of fully-connected layer
**b**: Bias of fully-connected layer
${\mathcal{B}}$: General differential operators that define boundary conditions
${\mathbb{R}}$: Real vector space
**s**: The first moment estimations
**r**: The second moment estimations
R^2^: Determination coefficient for performance characteristics
$L_{1}$: Relative field norm-1 error
$L_{2}$: Relative field norm-2 error
${\mathbf{W}}$^*^: The optimal weight of fully-connected layer

## Greek symbols

${\mathbf{\mathcal{D}}}$: Sampling space of dataset
${\mathbf{\mathcal{N}}}(\bullet)$: Nonlinear partial differential equations operators
$\Theta$: Learnable parameter set
$\Theta^{*}$: Optimal learnable parameters
α: Thermal diffusion coefficient
$${{\varvec{\upxi}}}$$: Input vector
$${\mathcal{L}}(\bullet)$$: Loss function
$${{\varvec{\Omega}}}$$: Computational domain
*ρ*: Density (kg·m^−^^3^)
$$\lambda$$: Thermal conductivity (W∙m^−^^1^∙K^−^^1^)
$${{\varvec{\upxi}}}^{\prime}$$: Normalized input
*μ*: Dynamic viscosity (Pa·s)
$\delta$: A small constant value for numerical stability
$\varphi$: Volume fraction of nanofluids (%)
*δ*: Groove relative depth
${\mathbf{\theta^{\prime}}}$: Normalized design variables
**θ**: Variable parameters
**σ**** (·)**: Active function operator
$$\beta$$: Attenuation coefficients
${{\varvec{\upeta}}}$: The output tensor of fully-connected layer

## Subscripts

n: Nanofluids
p: Nanoparticle
b: Base fluid
c: Mass conservations
bottom: Bottom wall
in: Microchannel inlet
out: Microchannel outlet
e: Energy conservations
g: Governing equation
mx: Momentum conservations along x coordinate
my: Momentum conservations y coordinate
top: Top wall
